# The School-to-Guardian Pipeline: Understanding the Medical and Social Implications for Individuals with Significant Disabilities

**DOI:** 10.1007/s11920-026-01718-9

**Published:** 2026-09-09

**Authors:** Jessie C. Green, Andrew Buck

**Affiliations:** Department of Psychiatry and Behavioral Health, Nisonger Center, College of Medicine, The Ohio State University Wexner Medical Center, 371 McCampbell Hall 1581 Dodd Drive, Columbus, OH 43210 USA

**Keywords:** Guardianship, Supported decision-making, Disability, School-to-guardianship pipeline

## Abstract

**Purpose of Review:**

As children move from being a minor to 18 and over as adults, the decision-making power shifts from the parent to the now adult child. That is unless legal guardianship is sought. One of the first encounters parents begin to really process the ramifications of this change is at school during individualized education program (IEP) transition planning meetings which begin for students with disabilities as early as 14 years in some states and school systems and required by federal law to occur by 16 years old. Local education agencies, or school districts, are required to inform students and parents of the shift in responsibilities when the child reaches the age of majority of 18 years old. This paper provides alternatives to guardianship and explores ways in which healthcare providers can support their patients who may be exploring decision-making options at 18 years old.

**Findings:**

Teachers and other school personnel often provide information that encourages parents to seek guardianship, pushing yet another student into the school-to-guardianship pipeline. Guardianship is not always the best option, yet it is often recommended, especially for transition-aged youth and young adults with significant disabilities. Healthcare providers are sometimes part of these school meetings, but more often, medical professionals provide assessment results to inform an individual’s need for guardianship to the courts.

**Summary:**

Healthcare providers should comprehend the different ways in which individuals with significant disabilities make decisions upon reaching adulthood. This knowledge should include a clear understanding of the implications and consequences of a court taking away partial or full legal decision-making capabilities from a young adult with a significant disability.

## The School-to-Guardian Pipeline: Understanding the Medical and Social Implications for Individuals with Significant Disabilities

As we come of age in the United States, it is assumed we become legally autonomous. An 18-year-old can now sign all the papers, vote in elections, obtain credit cards, and even get their own cell phone. That is unless you have a significant disability. The pipeline to guardianship starts often with the schools [1].

Alonso is a 17-year-old student with Down Syndrome. He is on an Individualized Education Program (IEP) to receive specialized services and supports at school. At this point, he is not receiving any other governmental supports (e.g., Medicaid, Developmental Disability state agency support). It is time for his annual review of the IEP, in which his mom, teachers, and school representatives are all present. Alonso has had the same IEP case manager for 4 years now, which has helped mom feel comfortable with the school and trust the case manager as a reputable resource as he transitions into adulthood.

At the annual review, things are going well until they reach the section titled Transfer of Rights at Age of Majority. This section of the IEP informs parents or legal guardians that the legal rights of the child transfer from the parents to the student unless guardianship is obtained. The school team insists Alonso’s mother take legal guardianship before he turns 18. Alonso’s mother is now concerned about implications despite him holding an afterschool job, actively participating in their church’s youth group, and gaining a driver’s license. He even went on a youth mission trip last summer without familial support. Later that evening, she sets in motion a plan to obtain guardianship.

The case above illustrates the role schools have in what is known as “the school-to-guardianship pipeline.” It puts school personnel in a place of authority on guardianship often without providing families with any other less restrictive alternatives to guardianship [1, 2] or no guardianship at all. While this pipeline may start in school, it is impactful for medical professionals. Guardianship takes away the ability for these young adults to make basic life decisions including what medical care they want to receive.

## Historical Relevance

Modern U.S. history has not always included individuals with disabilities in the everyday fabric of society. The 19th century placed people with disabilities into institutions with the intention of reentry into the community, but the late 1800’s institutions began to be a place to hide people with an intellectual disability who were seen as “dangerous to society” [3]. The 20th century led to what is known as the eugenics movement.

The eugenics movement often names scientist Sir Francis Galton as the father because he sought after a more perfect human race [3]. Eugenics is defined by the Merriam-Webster dictionary [4] as “the practice or advocacy of controlled selective breeding of human populations (as by sterilization) to improve the population’s genetic composition.” As such, segregated institutions began to sterilize people with disabilities [3]. Later, IQ testing was developed as a tool for removing a perceived threat to society by those with below average IQs. The primary diagnostic tool used today to diagnose intellectual disability is still IQ testing [5].

Disability activists have worked hard to uphold rights for people with disabilities. The 2006 United Nations Convention on the Rights of Persons with Disabilities is the first worldwide treaty to acknowledge disability. Its purpose is to “promote, protect and ensure the full and equal enjoyment of all human rights and fundamental freedoms by all persons with disabilities and to promote respect for their inherent dignity […], individual autonomy, including the freedom to make one’s own choices, and independence of persons” [6].

Civil rights law, Sect.  504 of the Rehabilitation Act of 1973, protects people with disabilities in many settings, including in healthcare facilities that receive federal financial assistance [7]. Section  504 prohibits discrimination based on disability, provides for reasonable accommodations to ensure equal access to care, prohibition of denial of services based on disability, and provides enforcement and complaint abilities.

Guardianship has long been the gold standard used to “protect” individuals with the most significant disabilities, often in tension with autonomy of the individual. Guardianship is a legal process completed at the state level which removes decision making from the individual transferring it to someone else, often a family member, but not always. Guardianship should be a final option, but often, it is used because of fear of compromised safety or possible assumptions about the person’s ability to make decisions [8]. One of the issues with guardianship is that there is not a consistent data collection system and so it is hard to true prevalence of the use of guardianship across the United States [1]. Uekert and Van Duizend based a “best guess” estimate off four states to be at approximately 1.5 million Americans are currently under full guardianship [9]. This estimate is also inclusive of all adults under guardianship, not just adults with an intellectual and developmental disability.

The medical model of disability has its roots in psychiatry [10]. Psychiatrist, Dr. Thomas Szasz argued mental health is not the same as a deficit, impairment, or limitation of a body system or an inherently pathological function that requires treatment through medical intervention [11] and therefore should not be used the same way in the context of the law [10]. Overall, the medical model posits disability to be a negative human condition and experience; often one to be cured, fixed, or normalized. Other models of disability exist, including the social model, the identity model, cultural model, and human rights model [12].

Rooting medical treatment in a framework in which there is a problem to fix or cure perpetuates stereotypes that people with intellectual disability are more vulnerable than the average person. This vulnerability promotes the idea that these individuals lack competence in all areas of their life, and therefore need to be protected, often with guardianship. Health care professionals often provide assessment and statement of individual capacity for an individual to make decisions as part of the legal guardianship process [13, 14].

### Educational Context

The case above illustrates what is known in special education as the school-to-guardianship pipeline and is typically articulated during the individual educational program meeting when educational professionals articulate to families via a necessary signature that when a student reaches the age of majority, the student will be responsible for all of their educational decisions moving forward. This is a point where educational professionals who are not trying to cause harm, provide legal advice without the full knowledge of the implications. While teachers are trying to help and meet mandates of IDEA, teachers are not trained legal experts and should not be giving advice to families on guardianship, rather they should be providing documentation and resources [8] for the options families to explore further.

Families want to provide protection to their loved one with a disability [8] and often feel the pressure of time when coming up against that 18th birthday, and act quickly and in the moment to obtain legal guardianship. This could also be a time when these same students are seeing pediatric doctors and specialists who are beginning to talk to parents about the transition from pediatric to adult health care systems.

## Consequences of the Pipeline

Automatically steering families down the guardianship pipeline does not come without consequence. The individual losing their guardianship as an adult can experience a loss of autonomy and have an impact on their well-being. Werner and Chabany found parents who had guardianship over their adult children continued to do what they always did in their parental role, often “blur[ing] the boundaries between tasks related to parenting and those related to guardianship.” [15] Parents also reported not having enough knowledge of what guardianship means and does not mean, as well as what other options may be available [8]. Warner and Chabany also found that individuals under legal guardianship would often go along with the decisions made for them by their guardian, but sometimes, the guardians would take decision making power too far [15].

Employment outcomes for individuals with guardianship has been found to not be as strong. Nord et al. found in an analysis of National Core Indicators outcomes, people with guardianship were less likely (odds ratio = 0.75) to have an employment goal in the service plan [16]. Without an employment goal in the service plan, individuals will not receive support services to prepare, look for, obtain, and maintain employment. For individuals with an employment goal in their service plan, there was not a significant difference in the types of employment (i.e., community, group, or facility) people held based on their guardianship status.

Regardless of guardianship status, quality of life measures continually show, individuals who are more autonomous in life activities have a higher quality of life [17, 18]. This does not mean that individuals with or without disabilities must be fully independent but rather have the tools to access necessary support to accomplish their goals.

Undoing guardianship is rare and likely going to be complicated to accomplish. Thus, entering into guardianship, should be considered thoughtfully rather than simply as a given next step for the individual with a disability. In certain circumstances, legal guardianship may be necessary; however, guardianship should be a last resort option after exploring less restrictive alternatives. There are considerations other than guardianship for people who need an added level of support.

## Alternatives to Guardianship

Guardianship implementation can vary from state to state. There are several alternatives to full guardianship used in healthcare settings, including Power of Attorney, advance directives, and Supported Decision-Making. Every situation is going to be unique, warranting careful consideration when the revocation of decision-making power is in question.

Power of Attorney does not typically involve the courts and is when the person gives the power to another person under their own volition, unlike with guardianship [19]. Advance directives operate similarly to a power of attorney allowing the individual to lay out what they wish to happen ahead of time.

## Supported Decision-Making

The 12th Article of the Convention on the Rights of Persons with Disabilities states “parties shall recognize that persons with disabilities enjoy legal capacity on an equal basis with others in all aspects of life.” This has started an international movement to use a supported decision-making (SDM) model. In the United States, nearly 40 states recognize supported decision-making through legislation [20]. States have legal frameworks, requirements for the courts to consider SDM, legislation for SDM as a healthcare “reasonable accommodation”, SDM built into high school transition planning, and SDM as a tool to the prevention of organ transplant discrimination [20].

SDM is defined by the National Resource Center for Supported Decision-Making as “a model for supporting people with disabilities, often cognitive disabilities, to make significant decisions and exercise their legal capacity. Specific decisions are addressed, weighed and concluded by the person with disability, while drawing on the support of (i) a network of people; or (ii) an individual.” [21].

Supported decision-making has many benefits, including the individual making an informed decision for themselves, increasing autonomy, enhancing self-determination [22], and steps toward increasing natural support networks [23].

Several U.S. states recognize supported decision making as a viable path for individuals with disabilities to make their own choices. In such states, a supported decision-making agreement is often, but not always part of the decision-making process. These agreements lay out who the agreement supports, who will support the adult with a disability, what topical areas the supporter may help to make decisions on, and what personal information (e.g., health, identifying, educational) the supporter may have access to. Agreements are often signed and notarized. An adult with disabilities may have multiple supported decision-making agreements with many different people based on the topic of support provided by each person or team.

## Policy and Practice Recommendations

When faced with a family seeking support of guardianship, it is imperative that as a clinician, you understand the reason for guardianship as well as the level of guardianship being sought. This could be an opportunity to advocate for alternatives to guardianship that are not as restrictive as full guardianship. This is also a time to screen for abuse, particularly financial abuse. Most importantly, this is the time to engage the individual who may lose guardianship in the decision-making process.

Several considerations should be made to ensure policy and practices are employed in favor of a supported decision-making model. This includes involving support personnel before an individual turns 18 in ways that empower the individual with a disability, rather than defaulting to the support person. Continuing this model after 18 years old is beneficial regardless of guardianship status.

It is essential to provide information on SDM in advance of an individual turning 18 as this gives time to practice SDM in medical settings early. Execution of SDM in medical settings at 18 will be smoother as everyone involved is prepared. This might look like the parent of a teen patient waiting in the waiting room like other parents during the appointment. Rather than springing this on the parent, notice could be given at one appointment on the move to provide the teen with a disability more independence in their medical appointment. Additionally, moving to a consult model at the end of the appointment can support building medical independence. Seeking consent from the patient throughout the appointment in many ways (e.g., to touch their body, to check in with the parent) will help to build decision-making and autonomy for the individual with a disability [24]. One last strategy could be to check for understanding frequently during an appointment.

Broadly, it is important for healthcare professionals to have training on disability rights and guardianship. In a study by Barjas-Ochoa et al., 77.5% of medical residents had no formal training provided by their medical school or residency [13]. These rights are provided to anyone with a disability, whether that is a staff or colleague, patients, visitors, and yourself. Disability rights are managed differently based on the stakeholder; however, it is important from the onset of one’s career to understand the impact across settings.

Additionally, for those practicing in pediatric populations, steps should be taken to understand the education implications of healthcare. For instance, scheduling staff can have a folder available with all the local school district academic calendars to ease scheduling conflicts. This may mean obtaining a Release of Information to do this work. Taking the release of information one step further may mean a practitioner needs to do a check for understanding that the individual understands the permission they are providing. This could look like an individual answering a few specific questions (e.g., Who are you giving permission for me to talk with? What are you giving me permission to talk about? ) or asking the person to explain the ROI form to you. This could be done with each new decision to ensure continued understanding.

Advocating with large health systems, advocacy organizations, and professional organizations for increased acceptance of the use of alternatives to guardianship could look like letter writing, testifying, or increasing knowledge of SDM practices among colleagues. This can be done locally (e.g., within practice), at a state level, or beyond.

Increasing the collaboration among medical, education, and legal professionals strengthens the scope of advice offered by interdisciplinary teams to disabled students and their families while ultimately supporting autonomous and well-informed decisions. Collaboration by healthcare professionals can take many forms, including participation in IEP and 504 meetings, providing extended information on a particular medical diagnosis, or giving time to consult with educators or legal professionals on impacts of guardianship versus a supported decision-making model.

### Future Directions for Research and Policy Change

Policy may need to be amended to ensure that billing and operational practices allow time for healthcare practitioners to attend meetings, gather and deliver thorough information, and to consult with educational or other team members. Dedicated time for these activities will extend the care students are receiving and increase quality of life.

Limited data on what type of involvement parents and guardians want to have in their children’s lives after reaching adulthood [8], what information is necessary to share with a parent or guardian to aid in treatment, and if there is an impact on overall health when the parent or guardian is involved. On the other hand, researchers are beginning to work with and listen to adults with lived experience share what they want from mental health providers [25].

Research on the supported decision-making model, including in medical settings, needs to continue focusing on successes, generalizations to other settings, and measuring how individuals are becoming better self-advocates. Quality-of-life outcomes should be measured in relation to SDM to better understand the impact of SDM on quality of life for people at risk of losing their legal autonomy.

## Conclusion

As part of the pediatric to adult healthcare transition process, guardianship and its alternatives, such as supported decision-making, should be shared with individuals and their families. Having information available early on SDM helps to pause, if not bypass the school-to-guardianship pipeline.

Terminating guardianship is a long, difficult process that often leaves the individual with a disability in at least a partial guardianship situation [26]. Determining how many guardian terminations are filed and granted, and the reasons for both terminations and denials, is necessary to further help professionals (e.g., healthcare, educational, legal) who are providing documentation and advice on alternatives to guardianship.

Navigating guardianship with families introduces complex legal and possible ethical challenges but is not a topic medical professionals should stray away from. Rather, lean into the opportunity you have to lift up a person with a more significant disability to be more autonomous with their decision-making power and increasing their quality of life.

## Key References

*Barajas-Ochoa A, Mackie TI, Fofana B, Rosen Valverde JN. On legal guardianship: An exploratory assessment of knowledge, attitudes and practices of resident physicians. Medical Teacher. 2024;46(3), 399–405. https://doi.org/10.1080/0142159X.2023.2256965
  - ○ This study is important in showing additional training is needed for medical students to fully understand guardianship, its alternatives, and implications of guardianship in their clinical practice.
*Minkoff RA. Guardianship knowledge amongst caregivers: A qualitative study. Journal of Social Service Research. 2022;48(5), 656-671. https://doi.org/10.1080/01488376.2022.2101583
  - ○ This study is important as it examines the perspectives of caregivers for adult children with significant disabilities. Results revealed that caregivers lack understanding of guardianship and alternatives, and obtain guardianship due to low expectations, fear, and school encouragement.
*Walters CB, Smith-Hill R, Plotner AJ, Springate A. Special education professional perspectives on challenges to supporting youth with disabilities and their families into legal adulthood. Education and Training in Autism and Developmental Disabilities, 2025;60(1), 35-48. https://doi.org/10.1177/215416472506000104
  - ○ This study sought the perspectives of special education professionals, and results found they lacked awareness along with families lacking knowledge of guardianship and alternatives, and families having low expectations of their children with disabilities resulting in a perceived need to obtain guardianship.

## Data Availability

No datasets were generated or analysed during the current study.
